# Congenital cytomegalovirus infection as an important cause of infantile cholestatic jaundice: a case report

**DOI:** 10.11604/pamj.2020.36.106.20577

**Published:** 2020-06-19

**Authors:** Olufunmilola Olubisi Abolurin, Idowu Odunayo Senbanjo, Adesola Olubunmi Adekoya, Emmanuel Damilare Ajibola

**Affiliations:** 1 Department of Paediatrics, Babcock University Teaching Hospital, Ilishan, Ogun State, Nigeria,; 2 Department of Paediatrics and Child Health, Lagos State University College of Medicine, Lagos, Nigeria

**Keywords:** Congenital cytomegalovirus, cholestatic jaundice, hepatitis, ganciclovir, infant

## Abstract

Infantile cholestasis has numerous causes and diagnosis can be difficult, especially in low-income countries where essential laboratory facilities are not readily available. This is a report of a baby who had severe conjugated neonatal hyperbilirubinaemia and deranged liver function tests, which posed a diagnostic dilemma before a diagnosis of congenital cytomegalovirus (CMV) infection was made. He was treated with Ganciclovir and responded well to treatment. He had no obvious associated neurologic manifestation of the disease and is presently been followed-up. This report highlights the challenges encountered in the diagnosis and management of the baby, as well as the favourable outcome with Ganciclovir therapy. The aim of the report is to increase the awareness of paediatricians and other stakeholders on congenital CMV infection in order to ensure early diagnosis and appropriate treatment of affected babies, with the ultimate aim of improving their prognoses and preventing the associated audiologic and cognitive sequelae.

## Introduction

Cytomegalovirus (CMV) is a member of the herpes group of viruses [1]. Human CMV infection is very common, with almost all individuals experiencing the infection at some point in their lifetime [1]. A significant proportion of persons acquire the infection during childhood, with the seroprevalence increasing gradually with age, to greater than 90 percent in the elderly [2,3]. Previous hospital-based reports among pregnant women in Nigeria had consistently revealed seroprevalence rates above 90 percent [4-7]. The infection is usually transmitted through contact with infected secretions, particularly saliva and urine, but can also be transmitted transplacentally from a mother to the foetus or through breastfeeding [2,8]. Typically, the infection goes unnoticed as it is usually asymptomatic. However, it can result in serious complications when acquired congenitally or perinatally, or in immunocompromised individuals [1,3].

A life-long latent phase usually follows CMV infection, with a risk of re-activation later in life. When a primary CMV infection or a re-activation in a previously infected person occurs, there is shedding of the infectious virus in the body fluids, with a risk of transmission to contacts [3,8]. Although most pregnant women have evidence of past CMV infection, only a few develop active infection during pregnancy, thus putting the foetus at risk. Foetal exposure is higher for women who have a primary CMV infection during pregnancy than for previously-infected women who experience a re-activation or re-infection [9,10]. About 10-15 percent of neonates with congenital CMV infection have symptoms/signs at birth [11,12]. Manifestations include premature birth, low birth weight, hepatosplenomegaly, thrombocytopaenia, jaundice, liver abnormalities, microcephaly as well as long-term audiologic and neurologic abnormalities [11,12]. While some survivors of congenital CMV infection may scale through without significant disability, others have life-long disabilities which significantly reduce their quality of life [12]. Congenital CMV infection, when symptomatic, is treated with antiviral drugs such as Ganciclovir and its pro-drug, Valganciclovir [13,14]. The most common adverse effects of Ganciclovir are neutropaenia, thrombocytopaenia, anaemia and phlebitis at injection sites [15,16].

We report a case of congenital CMV infection which manifested with severe conjugated neonatal hyperbilirubinaemia associated with deranged liver function tests. This report highlights the challenges encountered in the diagnosis and management of the baby, as well as the favourable outcome following treatment with Ganciclovir. The report is aimed at increasing the awareness of paediatricians and other stakeholders on the disease, so as to ensure early diagnosis, appropriate treatment and better prognosis.

## Patient and observation

Baby AO was admitted into the special care baby unit of the Babcock University Teaching Hospital, Ilishan, Nigeria shortly after caesarian delivery at a gestational age of 35 weeks, due to severe pre-eclampsia and severe intra-uterine growth retardation (IUGR). Birth weight was 1.2 kg and features of foetal malnutrition were evident in his physical appearance. At admission, he had good activity, but was dyspnoiec and hypoglycaemic; these abnormalities were managed appropriately. He was anicteric and had no palpable organ enlargement. Full blood count revealed mild thrombocytopaenia (platelet count 97x10^9^/L) and relative lymphocytosis, but haematocrit was normal (45%); serum electrolytes were within normal limits.

Jaundice appeared on the third day of life, followed by fever which was noticed the next day. Hyperbilirubinaemia was initially unconjugated with a total bilirubin of 120.3 μmol/L (7.1 mg/dl), with 14% conjugated fraction. The conjugated fraction increased to 21.9% within 24 hours, to 40.0% within 48 hours and had reached 67.0% by day six. The platelet count reduced gradually over the first two weeks of life to 46x10^9^/L. Haematocrit had also reduced significantly by then, necessitating blood transfusion. By day 14, the liver was palpably enlarged to three centimetres below the right costal margin and conjugated bilirubin had reached 74.7% of total, though the total serum bilirubin was not remarkably high (153.2 μmol/L). The liver enzymes were slightly elevated but clotting profile and serum proteins were normal. Initial hepatobiliary scan revealed no obvious abnormality. Tests for Hepatitis B, Hepatitis C, Syphilis and Human Immunodeficiency Virus were all negative. Feed tolerance was initially poor, with consequent weight loss. Serum bilirubin continued to rise all through the first month of life, eventually reaching 606.1 μmol/L (35.7 mg/dl). A repeat ultrasound done to exclude biliary atresia showed normal liver echogenicity with normal intra-hepatic ducts and vessels; the gallbladder was normal in size but its wall was thickened, hence cholecystitis was suggested. There were no sonographic features of biliary atresia.

The liver function parameters continued to worsen and alkaline phosphatase (ALP) rose to 1,627 IU/L. Hepatitis was considered, but its cause could not be ascertained. Reducing sugars were absent in the urine; alpha-1 antitrypsin assay was also requested but could not be done. Alkaline phosphatase continued to rise and eventually reached a peak of 3,090 IU/L. Other liver enzymes also remained above reference values. Baby had however started tolerating feeds and gaining weight. A repeat abdominal ultrasound revealed a contracted, poorly visualized gallbladder, and the triangular cord sign of biliary atresia could not be distinctively excluded. This heightened the suspicion of biliary atresia, and urgent surgical intervention was considered. Another scan however excluded biliary atresia.

Screening for cytomegalovirus (CMV) infection was then considered, which came out to be strongly positive with CMV IgM titre of 28.24 (Reference value 0.0 - 0.8). The mother also had serological evidence of CMV infection. The baby was given intravenous Ganciclovir for three weeks, followed by oral valganciclovir for another three weeks. Frequent intravenous cannulation was required during intravenous therapy due to Ganciclovir-induced phlebitis. Weekly monitoring of the full blood count was done during treatment, which revealed a decline in the neutrophil count, though there was no neutropaenia. Liver function parameters consistently improved during and after therapy, and had normalized by age 12 months. No obvious neurologic abnormality was documented throughout the period of treatment and the child´s developmental milestones have been within normal limits up till the age of 15 months when the child was last seen. Hearing test done by otoacoustic emission testing was normal in both ears, and visual test was also normal.

## Discussion

The potential complications of perinatal CMV infection makes it a dreadful problem in paediatric practice [12]. The manifestations of the disease, which are non-specific and vary widely, often simulate other clinical conditions; hence, many cases of perinatal CMV infection go undiagnosed in developing countries as a result of limited awareness of the disease as well as scarcity of diagnostic equipment [9,17]. The diagnostic dilemma encountered in the index case exemplifies the complexity that may arise in the evaluation and management of affected babies when the infection is not suspected early enough and screened for. Common causes of neonatal cholestatic jaundice include biliary atresia, idiopathic hepatitis, sepsis, alpha-1 antitrypsin deficiency and galactosaemia. These were considered in the index case before the diagnosis of CMV infection was made.

Gastrointestinal manifestations have been reported to occur frequently among infants with congenital CMV infection. Cheong *et al*. reported that up to two-thirds of infants diagnosed with congenital CMV infection developed gastrointestinal symptoms or signs [18]. Neurologic sequelae also occur frequently and may manifest as hearing loss, cognitive impairment, microcephaly, mental retardation or visual impairment. Permanent neurological sequelae were reported in 40-58 percent of infants whose CMV infection was symptomatic at birth and in about one-eight of those who had no symptoms at birth [11]. The baby in this report had no obvious neurologic manifestation of the disease and had normal visual and hearing tests several months after discharge. His good outcome might partly have been as a result of Ganciclovir therapy, which has been reported to reduce the risk of CMV-associated long-term sequelae and is also effective in the treatment of CMV hepatitis [13,14].

A high index of suspicion is required for early diagnosis of congenital CMV infection in countries like Nigeria where routine CMV screening is not practiced for pregnant women. Hence, babies with cholestatic jaundice, in whom the cause cannot be unraveled by routine tests, should be investigated for CMV infection. In the index case, the diagnosis of congenital CMV infection was made based on elevated CMV IgM antibody titres. Although CMV polymerase chain reaction (PCR) or viral culture is considered the gold standard for confirming congenital CMV infection, significant titres of CMV IgM antibodies are also highly suggestive of the acute illness and can be used for diagnosis when PCR is not available [1]. Some challenges were encountered during Ganciclovir therapy in the index case. Availability as well as the high cost of the drug posed an initial challenge. The frequent need for intravenous cannulation due to recurrent cannula failure as a result of drug-induced phlebitis also constituted a significant problem. Phlebitis is a common occurrence at Ganciclovir injection sites due to the high pH of the drug. This can be minimized by adequate dilution of the drug and slow intravenous infusion [15,16]. The co-operation of parents/caregivers can be enhanced by discussing these challenges with them before the commencement of ganciclovir therapy. Neutropaenia and thrombocytopaenia are well established side effects of Ganciclovir [15,16,19]. Regular monitoring of the full blood count during Ganciclovir therapy is therefore recommended. The index patient did not become neutropaenic in the course of treatment, although there was a gradual reduction in his neutrophil and platelet counts.

The diagnostic tests for CMV as well as the follow-up investigations and drug therapy for the disease would have constituted a huge financial burden, but for the health insurance benefit enjoyed by the family. Unfortunately, health insurance coverage is still very low in Nigeria, and the poverty rate is alarmingly high, such that many families cannot afford the high cost of medical treatment required in the predominantly out-of-pocket health-financing system that obtains in the country [20].

## Conclusion

CMV infection is a recognized cause of infantile cholestatic jaundice; hence infants in whom the cause of jaundice cannot be unraveled by routine tests should be investigated for CMV infection. This is important because the disease often results in life-long audiologic and cognitive sequelae which may be prevented by early diagnosis and appropriate treatment.

## References

[1] Ross SA, Novak Z, Pati S, Boppana SB (2011). Diagnosis of Cytomegalovirus Infections. Infect Disord Drug Targets.

[2] Staras SA, Dollard SC, Radford KW, Flanders WD, Pass RF, Cannon MJ (2006). Seroprevalence of cytomegalovirus infection in the United States. Clin Infect Dis.

[3] Bates M, Brantsaeter AB (2016). Human cytomegalovirus in Africa: a neglected but important pathogen. J Virus Erad.

[4] Akinbami AA, Rabiu KA, Adewunmi AA, Wright KO, Dosunmu AO, Adeyemo TA (2011). Seroprevalence of cytomegalovirus antibodies amongst normal pregnant women in Nigeria. Int J Women's Health.

[5] Ogbaini-Emovon E, Lofor PV, Oduyebo O, Ojide KC, Kalu IE, Joseph GA (2015). Incidence and risk of primary cytomegalovirus infection among pregnant women in Benin City Nigeria. Ann Biomed Sci.

[6] Anuma ON, Umeora OU (2016). Seroprevalence of cytomegalovirus antibodies among antenatal clinic attendees in Abakaliki, Nigeria. Afr J Med Health Sci.

[7] Yeroh M, Aminu M, Musa BO (2015). Seroprevalence of cytomegalovirus infection amongst pregnant women in Kaduna State, Nigeria. Afr J Clin Exper Microbiol.

[8] Ho M (1990). Epidemiology of cytomegalovirus infections. Rev Infect Dis.

[9] Britt WJ (2017). Congenital human cytomegalovirus infection and the enigma of maternal immunity. J Virol.

[10] Fowler KB, Stagno S, Pass RF (2003). Maternal immunity and prevention of congenital cytomegalovirus infection. JAMA.

[11] Dollard SC, Grosse SD, Ross DS (2007). New estimates of the prevalence of neurological and sensory sequelae and mortality associated with congenital cytomegalovirus infection. Rev Med Virol.

[12] Bale JF, Miner L, Petheram SJ (2002). Congenital Cytomegalovirus Infection. Curr Treat Options Neurol.

[13] Nassetta L, Kimberlin D, Whitley R (2009). Treatment of congenital cytomegalovirus infection: implications for future therapeutic strategies. J Antimicrob Chemother.

[14] Ozkan TB, Mistik R, Dikici B, Nazlioglu HO (2007). Antiviral therapy in neonatal cholestatic cytomegalovirus hepatitis. BMC Gastroenterology.

[15] Rossi S (2016). Australian Medicines Handboook.

[16] Alabsi S, Cunningham K, Willson R (2010). Guidelines for the Use of Ganciclovir.

[17] Olusanya BO, Slusher TM, Bopanna SB (2015). Prevalence of congenital cytomegalovirus infection in Nigeria: a pilot study. Paediatr Infect Dis. J.

[18] Cheong LY, Cowan FM, Modi N (2004). Gastrointestinal manifestations of postnatal cytomegalovirus infection in infants admitted to a neonatal intensive care unit over a five-year period. Arch Dis Child Fetal Neonatal Ed.

[19] Whitley RJ, Cloud G, Gruber W, Storch GA, Demmler GJ, Jacobs RF (1997). Ganciclovir treatment of symptomatic congenital cytomegalovirus infection: results of a phase II study. J Infect Dis.

[20] Adegboyega O, Abioye K (2017). Effects of health-care services and commodities cost on the patients at the primary health facilities in Zaria Metropolis, North Western Nigeria. Niger J Clin Pract.

